# Mechanisms of Action of Topical Corticosteroids in Psoriasis

**DOI:** 10.1155/2012/561018

**Published:** 2012-11-05

**Authors:** Luís Uva, Diana Miguel, Catarina Pinheiro, Joana Antunes, Diogo Cruz, João Ferreira, Paulo Filipe

**Affiliations:** Clínica Universitária de Dermatologia, Faculdade de Medicina de Lisboa, Av. Professor Egas Moniz, 1649-035 Lisbon, Portugal

## Abstract

Psoriasis is a lifelong, chronic, and immune-mediated systemic disease, which affects approximately 1–3% of the Caucasian population. The different presentations of psoriasis require different approaches to treatment and appropriate prescriptions according to disease severity. The use of topical therapy remains a key component of the management of almost all psoriasis patients, and while mild disease is commonly treated only with topical agents, the use of topical therapy as adjuvant therapy in moderate-to-severe disease may also be helpful. This paper focuses on the cutaneous mechanisms of action of corticosteroids and on the currently available topical treatments, taking into account adverse effects, bioavailability, new combination treatments, and strategies to improve the safety of corticosteroids. It is established that the treatment choice should be tailored to match the individual patient's needs and his/her expectations, prescribing to each patient the most suitable vehicle.

## 1. Introduction

Psoriasis is a lifelong, chronic, and immune-mediated systemic disease with preferential skin involvement, which affects approximately 1–3% of the Caucasian population [1, 2]. Psoriasis may appear at any age; however, over 75% of patients belong to a clear subgroup, that develops the disease before the age of 40 (type 1 or early-onset psoriasis) [3, 4]. The most common clinical variant is plaque-type psoriasis, characterized by erythematous scaly plaques, round or oval, variable in size, frequently located in scalp, lower back, umbilical region, intergluteal cleft, knees, and elbows [1, 5, 6]. As a clinically heterogeneous disease, psoriasis presents several degrees of severity and a wide array of presentations in different patients [7]. Approximately 80% of psoriasis patients have mild disease, with skin plaques usually covering less than 10% of the body surface area (BSA). However, some patients have moderate to/or severe disease, with greater than 10% of the BSA involvement [3, 6].

**Figure 1:**
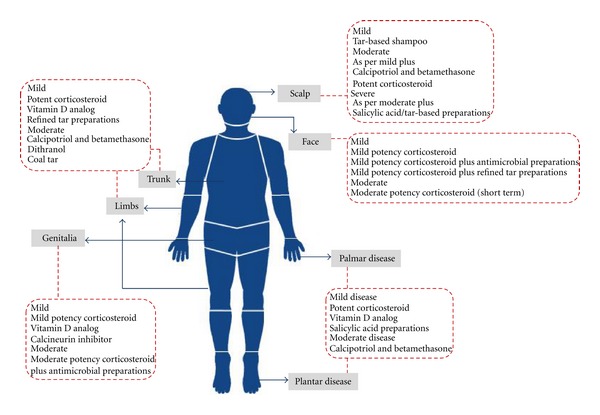
Topical therapy for management of psoriasis.

The different presentations of psoriasis require a variable approach to treatment and the current treatment concept advocates that the type of therapy prescribed should be appropriated to disease severity. Although there is a wide range of therapies available for the treatment of psoriasis, either systemic or topical agents, the use of topical therapy (Figure 1) remains a key component of the management of almost all psoriasis patients. While mild disease is commonly treated only with topical agents, the use of topical therapy as adjuvant therapy in moderate-to-severe disease may also be helpful and can potentially reduce the amount of phototherapy or systemic agent required to achieve satisfactory disease control.

Topical therapies available for mild-to-moderate psoriasis involve a great number of different agents, including [3, 7, 8]

- emollients;
- tars;
- dithranol;
- topical retinoids (Tazarotene);
- calcineurin inhibitors (pimecrolimus and tacrolimus);
- keratolytics (salicylic acid, urea);
- topical vitamin D analogues (calcitriol, tacalcitol, and calcipotriol);
- topical corticosteroids.

Since their introduction to dermatology, more than 50 years ago, topical corticosteroids have become the mainstay of treatment of various dermatoses including psoriasis, mainly due to their immunosuppressive, anti-inflammatory and antiproliferative properties, which makes this class of drugs an useful therapy for this immune-mediated disease [9, 10].

Although topical corticosteroids are an integral part of the psoriasis therapeutic armamentarium, limitations due to the occurrence of well-known cutaneous adverse effects such as atrophy, striae and/or telangiectases, and also potential systemic adverse events prevent their optimal long-term and extensive utilization. Therefore, strategies such as the weekend-only/pulse therapy regimen or combining topical corticosteroids with other topical agents may improve their efficacy and safety profile over longer periods [11, 12].

The purpose of the therapy is to reduce the extent and severity of psoriasis to the point at which it is no longer detrimental to a patient's quality of life. Treatment choice should always be tailored to match the individual patient's needs and his expectations. When employed under these circumstances, a topical treatment regimen is more likely to produce a satisfactory clinical outcome [3, 8].

## 2. Currently Available Topical Corticosteroids for Treatment of Psoriasis

Corticosteroids remain first-line treatment in the management of all grades of psoriasis, both as monotherapy or as a complement to systemic therapy. They are available in a wide range of preparations including gel, cream, ointment, foam, lotion, oil and spray, and a new and innovative vehicle (Table 1) [3, 16]. 

According to Cornell and Stoughton, we know that the vehicle can directly modify a preparation's therapeutic and adverse effects by changing the pharmacokinetics of the topical corticoid molecule. Therefore, the development of an improved vehicle for corticosteroids is at the forefront of dermatologic research [16, 17].

Although the decision of the agent depends on patient's choice, distribution of disease and local availability, bioassays comparing vehicles and corticoid molecules have demonstrated that ointments are the most effective, followed by creams and lotions. A recent study with clobetasol has suggested spray vehicle to be slightly more efficacious than other vehicles. Besides the important role of specific factors involved in the formulation of the spray, this greater efficacy may be due to increased patient compliance with an odorless, easy to apply, low residue, and elegant vehicle [3, 16, 18].

In 1985, Stoughton and Cornell classified corticosteroids potency according to their vasoconstrictive properties [3, 11, 17]. While in the USA there are seven potency groups, the UK considers four classes: mild (class IV), moderately potent (class III), potent (class II), and very potent (class I) [3, 11, 19, 20].

Lower-potency corticosteroids are particularly recommended to apply on the face, groin, axillary areas, and in infants and children, whereas mid- and higher-potency corticosteroids are commonly used as initial therapy on all other areas in adults. Superpotent corticosteroids are mainly used for stubborn, cutaneous plaques or lesions on the palms, soles, and/or scalp [11, 21, 22]. Regardless of the inexistence of studies to prove the assurance of topical corticosteroid use on the scalp beyond 4 weeks, in general, high-potency topical corticosteroids can be successfully and safely used. The reason for that safety is possibly due to the presence of dense vascularization and abundance of adnexal structures on the scalp that minimize the possibility of tachyphylaxis and side effects such as skin atrophy [23, 24].

As an initial therapy to achieve a faster improvement of lesions, in clinical practice, potent and superpotent corticosteroids are often used; however, they should not be used for more than 2 weeks and the patient should be under close surveillance [3, 11, 20, 25].

Psoriasis is a clinically heterogeneous disease, and its individual presentation can make the selection of the most appropriated treatment difficult. To overcome the variable nature of the disease and also the several options of treatment, there are currently two sets of guidelines from Germany [22] and USA [6] available for the different forms of topical treatment, which allow = a more effective therapy decision and to decide when patients move from topical to systemic treatment [7]. However, there are huge differences between recommendations from different countries [7, 9]. While German guidelines [22] recommend a combination of topical steroids with salicylic acid (broad combination is possible; care must be taken regarding steroid side effects), USA guidelines [6] suggest the use of topical steroids as monotherapy in mild-to-moderate psoriasis or in combination with other topical agents, UV light or systemic agents in moderate-to-severe disease [7].

## 3. Cutaneous Mechanisms of Action of Topical Corticosteroids

Psoriasis is an autoinflammatory and in some aspects an autoimmune disease of the skin. Both keratinocytes and leukocytes are actively involved in the immunopathology of the disease. Neutrophils, plasmacytoid dendritic cells (DCs), and CD11c^+^ (myeloid) DCs are present in psoriatic lesions as part of the innate immunity. Acquired immunity is diverted towards a T helper 1 (T_h_1) CD4^+^ cells-mediated response producing IFN-*γ*, TNF-*α*, and interleukin-2 (IL-2), along with T cytotoxic (T_C_) CD8^+^ cells [2]. Recently, T_h_17 cells have been suggested to be involved in the pathogenesis of psoriasis synthesizing IL-17A, IL-17F, and IL-22. T_h_17 cells' differentiation, proliferation, and survival are dependent on IL-6, TGF, IL-1*β*, IL-23, and IL-21. Particularly, IL23 is important for the pathogenicity at later stages of T_h_17 development. IL-23 and IL-12 share a common p40 subunit, which is covalently linked to either a p35 or p19 subunit forming IL-12 or IL-23, respectively. IL-23, secreted by activated myeloid dendritic cells, will drive T-cell differentiation toward Th17 subset and also release IL-12, inducing T_h_1 differentiation [26]. IL-17A leads to joint pathology due to its potential activity of inducing RANKL and its synergistic effect with IL-1*β* and TNF-*α*. Th17 cells produce IL-22, which have potent keratinocyte proliferative ability. IL-22, along with IL-17, induces STAT3 activation and cytokine/chemokine production, showing that way, an important role in the physiopathology of psoriasis. Anti-IL-17 monoclonal antibodies (AIN457 and LY2439821) may be useful in patients with psoriasis and autoimmune arthritis, as showed by successful experiments in animal models. Further clinical trials with these anti-IL-17 monoclonal antibody preparations in psoriasis and psoriatic arthritis are necessary [27].

Corticosteroids act in two different ways at the cellular level, divided into genomic and nongenomic pathways. 

The genomic pathway refers to the glucocorticoid receptor (GR) and to its activation by cortisol, subsequent receptor homodimerization, and binding to glucocorticoid-responsive elements (GREs). When the ligand is absent, the glucocorticoid receptor accumulates in the cytoplasm complexing with proteins, including the large heat shock proteins HSP90 and HSP70. But when the ligand binds to the receptor, this complex is disrupted and the GR migrates to the nucleus. Upon dimerisation of GR and binding to a palindromic promoter sequence, the glucocorticoid response elements, the transcription of genes with anti-inflammatory functions such as tyrosine amino transferase (TAT), phosphoenolpyruvate carboxykinase (PEPCK), IL-10, *β*-adrenergic receptor, IL-1-receptor antagonist, and dual-specificity protein phosphatase 1 (DUSP-1) are promoted. GC negatively regulates the expression of proinflammatory genes by transrepression, for example, cytokines, growth factors, adhesion molecules, nitric oxide, prostanoids, and other autacoids [28]. Further, coactivators or corepressors help modifying the structure of chromatin, enabling the DNA transcription [29]. The cortisol-glucocorticoid receptor complex may interact with nuclear factor-*κ*B leading to its transrepression (NF-*κ*B) [30, 31]. The latter mechanism apparently requires lower cortisol levels than the mechanism involving the GRE [32].

The nongenomic pathway takes membrane-bound receptors and second messengers into account, and it is responsible for the rapid effects of glucocorticoids that occur in a few minutes. This pathway does not require *de novo* protein synthesis and acts by modulating the level of activation and responsiveness of target cells, such as monocytes, T cells, and platelets [33, 34].

The glucocorticoid receptor is encoded by the *GR* gene, localized to chromosome 5q31-32 locus [32]. Posttranscriptional processing includes splicing of exon 9, yielding either *GRα* mRNA or *GRβ* mRNA [35]. Glucocorticoid receptor *α* isoform is responsible for the known actions of cortisol, whereas glucocorticoid receptor *β* isoform appears to play a regulatory role. An increase of the *β*/*α* isoforms ratio in a cell generates glucocorticoid resistance [36]. 

**Figure 2:**
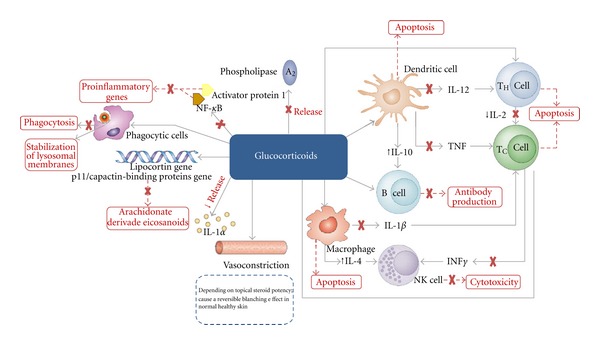
Anti-inflammatory, immunosuppressive, and vasoconstrictive effects of topical corticosteroids [13, 14].

Glucocorticoids possess numerous functions such as anti-inflammatory, antimitotic, apoptotic, vasoconstrictive and immunomodulatory functions. These properties are closely associated with their efficacy in the skin disease treatment (Figure 2) [37].

**Anti-Inflammatory Properties**

**Figure 3:**
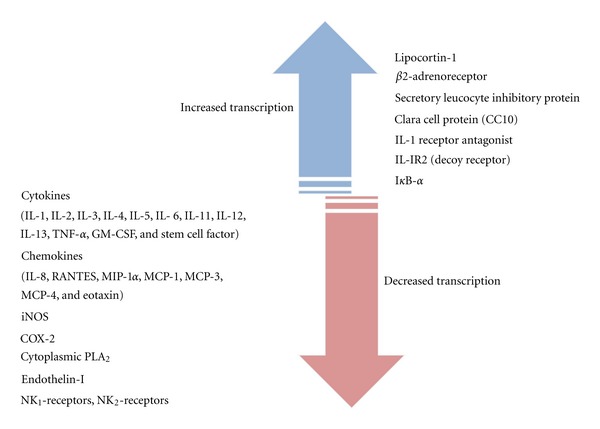
Action of glucocorticoids in gene transcription (adapted from [15]).

The inflammatory process is controlled by the glucocorticoids' activity, enhancing the transcription of anti-inflammatory genes and decreasing the transcription of inflammatory genes (Figure 3) [15].

Glucocorticoids induce the expression of annexin A1 (also known as lipocortin 1; encoded by ANXA 1) and ALXR (the annexin A1 receptor) by mechanisms still not known. Annexin A1 is a protein mainly located on basal keratinocytes of the basement membrane. Although in normal skin annexin A1 has been identified within cytoplasm, in diseased skin the intracellular localization of annexin A1 is apparently modified. In lesional psoriatic skin, annexin A1 appears only in the cell membrane, suggesting a translocation of the protein. This transition may occur to promote the binding of annexin A1 to phospholipids, therefore reducing the production of inflammatory prostanoids [37].

Annexin A1 inhibits phospholipase A_2_ (PLA_2_), thus blocking the synthesis of arachidonate-derived eicosanoids (prostaglandins, prostacyclins, leukotrienes, and thromboxanes) [32]. This blocking is furthered by the repression of glucocorticoid-mediated cyclooxygenase 2 transcription [38–41]. It remains unclear if the reduction of these substances levels come first and then plaque resolution, or if the normalization of prostanoid levels follows plaque clearance [37].

Exogenous and endogenous annexin A1 may regulate the innate immune cells activities controlling its levels of activation. Annexin A1 signals throw a formyl peptide receptor 2 (FPR2, ALXR in humans). Despite the activation of ALXR singnalling can occur by the annexin A1 autocrine, paracrine, and juxtacrine functions, the juxtacrine interaction seems to be the mechanism by which the anti-inflammatory process occurs. Concerning the innate response, it seems that the upregulation of the annexin A1 expression by leukocytes induced by glucocorticoids may be responsible for the inhibition of leukocytes response. Glucocorticoids also increase the secretion of annexin A1 by macrophages and the annexin A1 secreted by mast cells and monocytes, promotes the clearance of apoptotic neutrophils by macrophages. Endogenous annexin A1 is also released from apoptotic neutrophils and acts on macrophages promoting phagocytosis and removal of the apoptotic cells. The ALXR may be one mediator of this mechanism. Contrasting with the innate immunity, the adaptive immune system seems to act in a different way. Activation of T cells results in the release of annexin A1 and in the expression of ALXR. Although, glicocorticoids may reduce the annexin A1 expression within T-cell exposure as a consequence, there is an inhibition of T-cell activation and T cells differentiate into T helper 2 [42, 43].

Glucocorticoids induce expression of the MAPK phosphatase 1 (MKP-1). MAPK phosphatase 1 owes its anti-inflammatory properties to the interference in the MAPK pathway. MAPK phosphatase 1 dephosphorylates and hence further inactivates c-Jun (the terminal kinase in the MAPK pathway). The inactivation of MAPKs and also of MAPK-interacting kinase by MKP-1 is due to the inhibition of PLA_2_ activity mediated by glucocorticoids [32]. 

If glucocorticoids induce MKP-1 to suppress the inflammation, it seems that glucocorticoids resistance in some inflammatory diseases could be related to defects in the expression, or function, of MKP-1. It has been described in some inflammatory diseases that c-jun N terminal kinase and p38 activities are increased, becoming possible targets for clinical intervention. Possible mechanisms for glucocorticoids resistance may be associated with a failure in the inhibition of c-jun N terminal kinase and p38 because these kinases negatively regulate GR function. For example, an initial defect in glucocorticoids-induced MKP-1 expression/activity might increase MAPK activity, thus impairing the GR function. As a consequence of this failure, an increase in transcription of proinflammatory genes, or in the instability of the mRNAs, may occur. Alternative to the hyperactive MAPK pathway, a reduced number of activated GR within the nucleus or a lack of interaction with the basal transcription process may be a reason for steroid resistance. It can be relevant, concerning the glucocorticoid tachyphylaxia, whether these mechanisms can be implicated [44, 45]. 

c-Jun is a transcription factor recognized to form homodimers and heterodimers with c-Fos, the latter combination resulting in the activator protein 1 (AP-1). Both c-Jun homodimer and AP-1 heterodimer are associated with transcription of inflammatory and immune genes. There is also evidence of direct protein-protein interactions between the glucocorticoid receptor and c-Jun homodimers and AP-1 heterodimers, conferring to the nongenomic pathway of cortisol a large share of the anti-inflammatory action of glucocorticoids [31]. Other genomic mechanisms include the direct repression of the NF-*κ*B transcription factor by the glucocorticoid receptor. NF-*κ*B binds to DNA and induces transcription of genes encoding cytokines, chemokines, complement proteins, cell-adhesion, molecules and cyclooxygenase 2 [46], all associated with inflammation.

It is unquestionable that the expression and activity of several cytokines relevant to inflammatory diseases may be inhibited by treatment with glucocorticoids. These cytokines include IL-1, IL-2, IL-3, IL-6, IL-11, TNF-*α*, GM-CSF, and chemokines that “call” inflammatory cells to the site of inflammation, namely, IL-8, RANTES, MCP-1, MCP-3, MCP-4, MIP-1*α*, and eotaxin. Furthermore, the inflammatory process receptors, such as NK_1_ and NK_2_-receptors, are involved in the transcription of genes coding for these mediated inflammatory receptors by glucocorticoids activity. The glucocorticoids suppressive effects are related with inhibition of cytokine gene expression by inhibiting the transcription factors that regulate their expression, rather than binding to their promoter regions [15]. 

Regarding genomic and nongenomic pathways, it seems that the nongenomic pathway stands out powerful enough to mediate the anti-inflammation process by itself. In an experiment, the *GR* mouse gene was mutated so that the glucocorticoid receptor lost the ability to dimerize, and thus bind DNA. In these *GR* ^*dim*/*dim*^ mice, glucocorticoids were only allowed to act via the nongenomic pathway. A phorbol 12-myristate 13-acetate- (PMA-) mediated ear edema was then induced in both wild-type and *GR* ^*dim*/*dim*^ mice. Surprisingly, the edema was reduced in both strands of mice after administration of dexamethasone. Additionally, dexamethasone suppressed serum TNF-*α* and IL-6, and lipopolysaccharide (LPS)-induced transcription of TNF-*α*, IL-6, IL-1*β*, and cyclooxygenase 2 genes in both wild-type and mutant mice [47]. 

Nitric oxide (NO) has a relevant function in multiple systems modeling physiological and pathological processes in the skin, namely, vasodilation, immunomodulation, inflammation, and oxidative damage to cells and tissues. Thereby, NO represents another possible target for glucocorticoids. The synthesis of NO is dependent of the nitric oxid synthases (NOS), a family of enzymes with three isoforms, the constitutive endothelial eNOS, neuronal nNOS, and the inducible iNOS. While glucocorticoids restrain the induction of iNOS, they do not produce any effect over eNOS and nNOS activity.

It is thought that inhibition of NO by glucocorticoids occurs only in the presence of high NO levels, caused by inflammatory substances such as lipopolysaccharides or cytokines, in a similar way to the COX system. The nitric oxide synthases' inhibitors appear to be related with the NO role in erythema and oedema formation in psoriasis. Moreover, iNOS was found in lesional psoriatic skin [37, 48]. 

The modulation of mast cell numbers and activity has been suggested as an additional mechanism for the anti-inflammatory properties. These cells have numerous pro-inflammatory mediators, as histamine and prostaglandins, which are released, in response to mast cell degranulation. Thereby, we may obtain an anti-inflammatory action by inhibiting this mast cell reaction. The use of glucocorticosteroids diminishes the number of mast cell in the skin, which is responsible for reducing histamine content in the treated skin [37, 49].

**Antiproliferative Properties**

Another beneficial action of the topical glucocorticoids is their antimitotic activity, which has been suggested as providing positive results in the treatment of psoriasis, where cell turnover rate of the skin is substantially elevated. Studies on normal and psoriatic skin suggest that topical glucocorticoids decrease the number of epidermal mitoses. Dexamethasone may have an anti-proliferative effect over the A549 cell line, which is associated with an increase of annexin A1 [37]. It would be of interest to find out whether the antimitotic power of glucocorticoids is caused by these effects on annexin A1 in order to develop new therapeutic tools and diminish the skin thinning.

**Apoptotic and Antiapoptotic Properties**

Eosinophils and lymphocytes are also an aim of glucocorticoids therapy. These drugs decrease the survival of both types of cells, leading to programmed cell death or apoptosis. The apoptosis of eosinophils appears to be related with a blockade of the IL-5 and GM-CSF effects, of which eosinophils are dependent. On the contrary, glucocorticoids reduce apoptosis and enhance neutrophils survival [15].

**Vasoconstrictive Properties**

Although associated with an unclear mechanism, the vascular action has been proposed to be part of the anti-inflammatory effects of glucocorticoids, since there is a reduction in blood flow to the inflamed site. Vasoconstriction, also termed “blanching,” when related to skin surface, forms the basis of the standard assay for evaluation of the potency of topical glucocorticoids [37].

**Immunosuppressive Properties**

The regulation of several aspects of immune-cell function is also pertinent to the cutaneous function of glucocorticoids, conferring an additional benefit in treatment of dermal diseases. In addition to inhibiting humoral factors involved in the inflammatory response and the leukocyte migration to sites of inflammation, glucocorticoids interfere with the function of endothelial cells, granulocytes, and fibroblasts. Therefore, glucocorticoids commonly repress maturation, differentiation, and proliferation of all immune cells, including DCs and macrophages. Suppressing dendritic cells and macrophages, and consequently the production of T helper 1-cell-inducing cytokine interleukin-12 (IL-12), glucocorticoids generate a shift in adaptive immune responses from a T_h_1 type to a T_h_2 type. Furthermore, these drugs may amplify delayed-type hypersensitivity [13].

### 3.1. Systemic Adverse Effects of Topical Corticosteroids

Considering the broad array of interactions between glucocorticoids and specific and nonspecific molecular targets within the cell (Figure 3), it is expectable that prescribing corticosteroids may produce a wide range of undesirable adverse effects. This has led, in fact, to a “steroid phobia” among patients [50]. The adverse effects of glucocorticoids tend to be more severe with systemic rather than with topical treatment [51]. Nevertheless, glucocorticoid topical therapy for cutaneous and pulmonary (nasal administration) diseases is known to be associated with systemic adverse reactions [52, 53].

The impaired barrier function in psoriatic skin facilitates the cutaneous penetration of the topical corticosteroid independently from its potency. The concomitant vasodilatation in psoriatic vessels increases the possibility of topical corticosteroids to reach the systemic vessels. A large extent of body surface and long-term use of topical corticosteroids may conduct to a higher concentration of corticosteroids in the blood, leading to systemic side effects. The risk of systemic side effects associated with chronic topical corticosteroid use increases with high-potency formulations.

As anti-inflammation is one of the main goals in the treatment of psoriasis, it should be noted that the lack of immune function, a state of immunosuppression, brings about opportunistic infections that the human organism would otherwise efficiently deal with. Among these, there are infections caused by *Candida* spp., or reinfections caused by previously latent virus, like *Cytomegalovirus* [51]. Endogenous hypercortisolism may also account for these infections [54]. 

Overt cataract and glaucoma may also develop [55, 56], due to the effects that glucocorticoids have on the endocrine and cardiovascular systems. Glaucoma is a consequence of an increased intraocular pressure. Exogenous corticosteroids are not inactivated by 11*β*-hydroxysteroid dehydrogenase, so they actually activate the mineralocorticoid receptor allowing ENaCs to increase serum Na^+^ levels and causing hypertension [57]. Other glucocorticoids cardiovascular adverse effects include a hypercoagulability state and dyslipidemia [58, 59]. The correlation with the glucose metabolism is notorious, since glucocorticoids may aggravate previous diabetes mellitus [51]. Indeed, the United States of America's National Health and Wellness Survey (NHWS) identified psoriasis to be associated with cardiovascular risk factors such as hypertension, hypercholesterolemia, and diabetes [60]. Glucocorticoids promote hepatic gluconeogenesis [51]. Glucose-6-phosphatase, a key enzyme in the gluconeogenesis pathway, is encoded by the *G6Pase *gene. The *G6Pase* gene promoter includes a glucocorticoid-responsive element (GRE), this way augmenting the gluconeogenesis rate after glucocorticoid receptor activation [61]. Glucocorticoids concomitantly generate iatrogenic Cushing's syndrome and adrenal insufficiency [62]. High levels of glucocorticoids in the bloodstream imbalance the hypothalamus-pituitary-adrenal axis equilibrium and suppress the ACTH levels, as a result of a negative regulatory effect on ACTH release. It leads to adrenal cortex atrophy and, thereafter, to complications like hypogonadism, inhibition of growth, or osteoporosis [51]. 

Osteoporosis is a serious complication of glucocorticoid treatment, particularly when affecting trabecular bone [63]. The pathophysiology underlying osseous degradation is related to the upregulation of the receptor activator of nuclear factor kappa-B ligand (RANKL) mRNA by glucocorticoids, helping osteoclasts to differentiate and therefore degrading bone. On the other hand, osteoprotegerin (OPG) gene transcription is repressed [51].

Myopathy and muscle atrophy are also possible adverse effects of glucocorticoid treatment. It was found that proteolysis is augmented in myocytes, due to a glucocorticoid-mediated increase in the transcription of genes-encoding proteins linked to the ubiquitin-proteasome pathway [64].

Glucocorticoids may also aggravate previous psychiatric disorders [65]. Accordingly, the NHWS places depression as a comorbidity significantly associated with psoriasis [60].

### 3.2. Cutaneous Adverse Effects of Topical Corticosteroids

**Figure 4:**
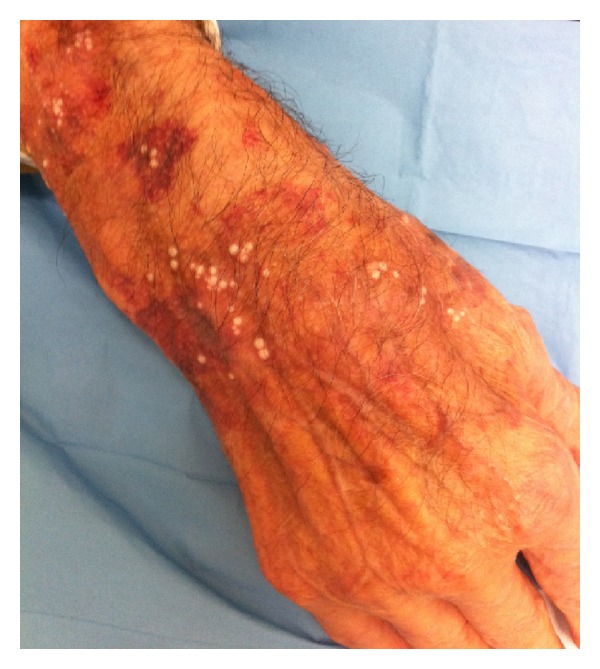
Purpura, milia and rubeosis steroidica induced by superpotent topical corticosteroids.

The very first contact that the patient has with topical corticosteroids is mostly through skin. Although corticosteroids help mitigate psoriatic lesions, cutaneous side effects are numerous and not rare. Skin atrophy, striae rubrae distensae and perturbed cicatrization are the most common. Hypertrichosis, steroid acne, perioral dermatitis, erythema, and telangiectasia may also occur. Erythema and telangiectasia together with skin atrophy may lead to permanent rubeosis steroidica (Figure 4). Hyperpigmentation is rarer than the above-mentioned adverse effects [51]. 

Glucocorticoids-mediated skin atrophy involves thinning of the epidermis and dermis (and even hypodermis), resulting in increased water permeability and, thus, in increased transepidermal water loss [66, 67]. The thinning is caused by a decreased proliferative rate of keratinocytes and dermal fibroblasts [68]. The origin of the decreased proliferation lies in collagen turnover. Transforming growth factor *β* (TGF-*β*) is a signaling molecule that, among other actions, promotes production of collagen, using Smad proteins as second messengers [69]. Activated GR negatively regulates Smad3 through a protein-protein interaction, in this way, blocking expression of the *COL1A2* gene, which encodes a type I collagen chain [70]. Type I collagen represents roughly 80% of the total share of skin collagen [51]. Therefore, glucocorticoids reduce collagen turnover through blocking of TGF-*β* actions. Coincidentally, TGF-*β* plays a central role in the epithelial-to-mesenchymal transition (EMT), an essential mechanism for cicatrization [71–73]. Glucocorticoids also diminish synthesis of epidermal lipids [67]. Furthermore, glucocorticoids reduce collagenases, which are part of the matrix metalloproteinases (MMPs) and tissue inhibitors of the metalloproteinases TIMP-1 and TIMP-2. Striae formation, which occurs in hypercortisolism and may occur after long-term topical treatment with glucocorticoids, may be explained by the skin tensile strength determined by type I and type III collagens [74–77]. The thinning of epidermis caused by glucocorticoids' long-term topical treatment appears also to be related with the repression of K5–K14 keratin genes, which are markers of the basal keratinocytes. Additionally, these drugs inhibit K6–K16 keratin genes, markers of activated keratinocytes, therefore promoting impaired wound healing. 

Special attention should be paid when applying topical corticosteroids in the presence of an infection, as there is a risk of exacerbation. Topical corticosteroids can inhibit the skin's ability to fight against bacterial or fungal infections. A common example of this inhibition is seen when a topical steroid is applied to an itchy groin rash. If this is a fungal infection, the rash gets redder, itchier, and spreads more extensively than a normal mycosis. The result is a tinea incognito, a rash with bizarre pattern of widespread inflammation [78]. 

Glucocorticoids' adverse effects are an obstacle to psoriasis treatment. Abolishing these reactions and at the same time maintaining glucocorticoids efficacy has been a challenge to researchers. Selective glucocorticoid receptor agonists (SEGRAs or, alternatively, dissociating glucocorticoids), nitrosteroids, and liposomal glucocorticoids are under development [79]. To this date, we still rely on conventional glucocorticoids.

## 4. Bioavailability of Topical Corticosteroids

The type of psoriasis and drug metabolism in the skin are the main factors that influence bioavailability of topical corticosteroids.

Alterations in the epidermal permeability barrier may contribute to psoriasis, as evidenced by the enhanced transepidermal water loss. Recently, an association between the gene of psoriasis and variations in the late cornified envelope gene loci has been confirmed, establishing a relation between an alteration of the permeability in the epidermis and the pathogenesis of the disease [80]. There is also the 500 Dalton rule for the skin penetration of chemical compounds and drugs, which states that molecules above that weight are not capable of crossing the stratum corneum [81]. For instance, topical tacrolimus (802 Da) is not effective in chronic plaque-type psoriasis but it is useful in psoriasis in the face or intertriginous areas, in pustular psoriasis [82], and when combined with descaling agents [83–86].

Skin acts as a barrier due to its physicochemical properties [87] and to the enzymes present in the keratinocytes (cytochrome P450 enzymes), which inactivate some topical corticosteroids and metabolize others in more active substances [88]. Corticosteroids are lipophilic and readily migrate through the cell membrane to bind the corticoid receptor thus forming dimers, which then migrate to the cell nucleus inducing the therapeutic effect by regulating gene expression [89]. Fluticasone propionate and methylprednisolone aceponate are very lipophilic, and due to that they have an increased bioavailability; but while the first one is hydrolyzed in an inactive substance, the last one is hydrolyzed by cutaneous esterase's in a more active metabolite [10].

One of the most important points to achieve the success in treatment is to choose the best corticosteroid formulation according to each patient. There is an array of manufactured vehicles including creams, ointments, lotions, foams, oils, gels, solutions, drops, shampoos, sprays, and tape; the efficacy rates between them are roughly comparable [90]. For example, scalp, foams, gels, or sprays may be more easy to apply, and so, a better result is expected. 

The vehicle has a therapeutic effect; scalp lipogel without active ingredients showed response rates of over 20% in scalp psoriasis [91]. Also a 15%–47% response to placebo was described with emollients in psoriatic patients, but it is already known that hydration improves signs and symptoms of psoriasis [6, 21]. 

Ointments are composed by more than 70%, of lipids, lipid-rich creams by 70% and creams only 15% to 25%. In contrast to what was assumed, a recent study with betamethasone with a low-lipid content formulation showed a higher efficiency than high-lipid concentrated creams and ointments, confirming the need of tailor therapies to individual patients and the impact of bioavailability of specific components of the vehicles. We can add specific ingredients to increase bioavailability; for instance, propylene glycol is a percutaneous absorption enhancer of hydrocortisone [10].

The volume of the prescription should be planned considering the frequency and the effective dose; the fingertip unit is used as a pattern for the topical agent required. Dressings are also used, enhancing the drug delivery, and this choice depends on local availability and patient preference [92].

When applied in a higher concentration, or multiple times a day, the levels of a corticoid (triamcinolone acetonide) in the stratum corneum of the skin, after 24 h, were the same as in a lower dose and a less frequent application [10].

Topical treatments in psoriasis should be specific to each topographic region, and steroids are absorbed at different rates in different parts of the body as follows:

- eyelids and genitals absorb 30%;
- face absorbs 7%;
- armpit absorbs 4%;
- forearm absorbs 1%;
- palm absorbs 0,1%;
- sole absorbs 0,05%.

Vitamin D analogues and low-potency steroids in a cream base are indicated for psoriasis of the face or flexures [93]. The scalp skin has very specific properties, it is covered by hair and sebaceous glands are abundant; therefore, a large proportion of the drug applied is wasted and adhered to the hair not having contact with the scalp. New vehicles are proposed to improve the treatment of scalp psoriasis such as calcipotriol-betamethasone dipropionate scalp lipogel [91], clobetasol propionate and betamethasone valerate foam [94], clobetasol propionate shampoo [95], and clobetasol propionate 0.05% spray [96].

Combined treatments with different biological targets are already accepted, usually having an additive or synergistic effect. These treatments act by adding the effects on different targets (T-cell functions, innate immunity, epidermal differentiation, and proliferation), reducing the side effects and managing recalcitrant lesions. Topical therapies can be used during the phase that the systemic treatment is suboptimal [10].

## 5. New Combination Treatment of Topical Corticosteroids

Combination therapy has emerged with the development of new noncorticosteroid preparations, but before the merge we have to make sure that the two combinations are compatible, synergistic, and safe. As an example, calcipotriol is just compatible with tar gel and halobetasol propionate preparations and it has a superior effect when combined with halobetasol ointment. Halobetasol decreased the irritant dermatitis caused by calcipotriol [97, 98]. Also ammonium lactate is compatible with hydrocortisone valerate and halobetasol propionate, and it has been shown to protect against skin atrophy [10, 99].

Salicylic acid, vitamin D analogues and retinoids, with different mechanisms of action, are usually combined with topical corticosteroids. Concerning polytherapy versus fixed-dose combinations, the last one requires less frequent applications and has a higher adherence from the patients.

### 5.1. Topical Vitamin D Analogues and Corticosteroids

These agents were found in the sequence of the discovery that oral vitamin D had a therapeutic effect on psoriatic plaques [100].

While vitamin D has mainly antiproliferative (epidermal) effects, corticosteroids have mainly anti-inflammatory (dermal) effects.

The vitamin D analogues correct epidermal hyperproliferation, abnormal angiogenesis, and keratinization and induces apoptosis in inflammatory cells by acting through vitamin D receptors present on keratinocytes and lymphocytes. They also modulate the decrease of IL-1 and IL-6 levels, the reducing of CD45RO and C8^+^ T cells. They inhibit the epithelial cell growth by increasing transforming growth factor-*β*1 and -*β*2 levels [101]. Many of these effects protect skin from the cutaneous atrophy caused by corticosteroids [102].

Vitamin D analogues and corticosteroids are the combining topical agents of choice in psoriasis showing a superior efficacy when compared with monotherapy [20].

The most common side effects are skin irritation, dryness, peeling, erythema, and edema, which can occur in up to 35% of the patients. Adverse effects will diminish along the time.

At the moment, three vitamin D analogues are approved for the treatment of psoriasis: calcitriol, calcipotriol, and tacalcitol.

The corticosteroids effects can be diminished by administrating calcipotriol 0.03% once daily in the morning plus betamethasone valerate once daily in the evening [103].

Calcipotriol is the vitamin D analogue most widely accepted for the combination therapy with corticosteroids, although not all corticosteroids can be mixed with calcipotriol due to incompatibilities [97]. A combined ointment with calcipotriol and betamethasone dipropionate is already being used and showing good results, giving to the patient's skin stability and optimal delivery of both substances. Cutaneous atrophy caused by this ointment is similar to the corticosteroid alone during a 4-week treatment period [104, 105]. Recent data says that the combination has proved to be superior in efficacy than the individual components alone [106].

This combination of calcipotriol and betamethasone dipropionate is approved for use in trunk and extremities, but it is not recommended for face, intertriginous areas, and scalp. Although this combination has now been developed as an oily lipogel indicated for scalp psoriasis, showing the same efficacy, safety, and tolerability as the ointment [91, 107].

### 5.2. Topical Salicylic Acid and Corticosteroids

Salicylic acid is a topical keratolytic used in the treatment of a variety of papulosquamous lesions such as psoriasis. Its mechanism of action is still unclear, but it is believed to act by inducing disruption of keratinocyte-keratinocyte binding and softening of the stratum corneum by decreasing its pH [108].

Salicylic acid has a filtering effect reducing the efficacy of UVB therapy, so it should not be applied before treatment. Due to scarce data, it should not be applied during pregnancy [3].

Usually, salicylic acid is safe; however, with long-term use in large skin areas, systemic salicylic acid toxicity can occur [108].

Studies with tritiated triamcinolone acetonide, desoximetasone, and hydrocortisone 17-valerate showed that salicylic acid enhance the efficacy of these corticosteroids by increasing their penetration in skin. This faster penetration of corticosteroids in skin does not occur when mixed with other ingredients such as camphor, menthol, phenol, or urea.

Fixed-dose combinations such as salicylic acid and betamethasone propionate or salicylic acid with diflucortolone are already available in some countries and they show similar efficacy. They both appear to be efficacious and well tolerated during short-term period treatment of plaque psoriasis and their use is recommended for limited areas of skin: for thick, scaly, and psoriatic plaques [10]. 

### 5.3. Topical Tazarotene and Corticosteroids

Tazarotene was the first topic retinoid found to be effective for mild-to-moderate psoriasis and it is available in cream or gel form.

Tazarotene is a retinoid derivate which binds the retinoic acid receptor (RAR) in a class-specific manner, preferentially binding RAR-*γ* and RAR-*β* than RAR-*α* [109]. This regulation of transcription result in reduced keratinocyte proliferation, normalized keratinocyte differentiation, and decreased inflammation. Its protective role against cutaneous atrophy from corticosteroid induction, may be important already shown by the retinoid tretinoin [110]. 

This retinoid may cause skin irritation in up to 30% of users [111], and this irritation was more pronounced in patients receiving tazarotene plus corticosteroids than in those receiving calcipotriol [112].

Retinoids may reduce UVB tolerance, and tazarotene has proven to be more efficacious than UVB alone [113].

As the systemic retinoids, tazarotene is contraindicated in pregnancy.

It is not indicated to prescribe tazarotene mixed with corticosteroids. Although tazarotene showed to be chemically compatible with a number of topical corticosteroids, no experiment testing over two weeks of treatment has been performed [114]. 

## 6. New Strategies to Improve Safety of Topical Corticosteroids

Taking into account one of the most important features of psoriasis, its chronic nature, the therapeutic approach should be prolonged, which makes it challenging to use high-potency topical corticosteroids safely in long-term management of the disease [3, 11].

**Figure 5:**
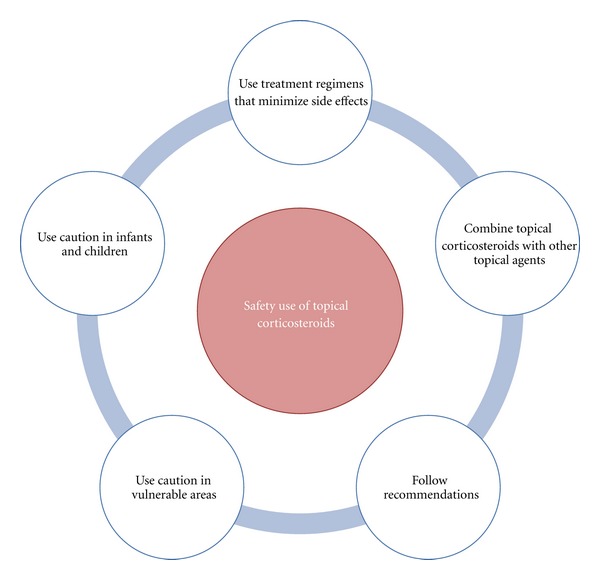
Strategies to improve safety for long-term use of topical corticosteroids in psoriasis.

Therefore, therapy should be monitored by a competent healthcare professional to limit the risk of cutaneous or systemic side effects, and some general principles should be followed to minimize these effects (Figure 5). The untoward effects of topical corticosteroids have been well documented and can be widely categorized as local (atrophy, telangiectasia, striae distensae, folliculitis, acne, and purpura) or systemic (hypertension, osteoporosis, Cushing's syndrome, cataracts, glaucoma, diabetes, and avascular necrosis of the femoral head or humeral head) [3, 11].

### 6.1. Using Treatment Regimens That Minimize Side Effects

In order to reduce side effects for long-term use of topical corticosteroids, a number of new therapy regimens have been studied. One of them is weekend or pulse therapy where three consecutive doses of corticosteroids at 12 h intervals are given on the weekends, following a successful initial cleared or almost cleared therapeutic response to daily potent topical corticosteroids application [115, 116].

### 6.2. Combining Topical Corticosteroids with Other Topical Agents

The combination of topical corticosteroids with other topical anti-inflammatory agents, as steroid-sparing therapies, can result in an improvement of efficacy with less side effects. Sequential therapy with higher-potency corticosteroids in combination with a vitamin D analogue such as calcipotriene can increase short-term efficacy and decrease side effects in long-term treatment [117].

Another successful combination is topical corticosteroids and tazarotene, which has improved efficacy compared to tazarotene therapy alone [114, 118, 119]. Whilst corticosteroids maximize efficacy and minimize toxicity of tazarotene, this drug reduces the development of corticosteroid-induced cutaneous atrophy [110].

Salicylic acid can also be applied in combination with mild-potency corticosteroids increasing the skin penetration [118].

Besides the combination with other topical agents, corticosteroids are often used in combination with UVB phototherapy, traditional systemic agents (acitretin, cyclosporine, and methotrexate), and biological agents [11].

### 6.3. Following Recommendations of Usage

The topical corticosteroid recommendations suggest, for the most corticosteroids, 60 g, as a maximum dosage per week, and for some superpotent corticosteroids 50 g per week. However, there are particularly cases in which patients need to exceed what the package insert recommends, and in those circumstances the possibility of systemic absorption must be considered.

For thick areas that are more resistant to steroid side effects such as the palms and soles, limited occlusion may be necessary and would require close followup, but for more prone areas such as the axillae, groin, and face, occlusion is more probable to result in adverse effects [11].

### 6.4. Considering Use in Children

Children are more susceptible to systemic side effects, like hypothalamic-pituitary-adrenal axis suppression, compared with adult patients, owing to their greater body surface area-to-weight ratio. Accordingly, lower-potency corticosteroids are frequently applied in infants and children [3, 11, 120].

### 6.5. Considering Use in Vulnerable Areas

The face and the intertriginous areas are particularly sensitive to untoward effects. For that reason, the protracted use of corticosteroids, even with lower potency, can be associated with telangiectasia on the face and formation of striae on intertriginous sites such as the groin, axillae, or under the breasts [11].

Even though safety of topical corticosteroids and other topical treatments has been recently reviewed, additional studies of topical corticosteroids are imperative. 

## 7. Comments

Mild-to-moderate psoriasis can be controlled with topical therapy; however, topical therapy should be administrated with adjunctive therapy in severe and extended psoriasis.

Glucocorticoid research is an ongoing process with the development of hyperselective therapeutic agents acting at different stages of the psoriasis inflammatory response. One of the most desired targets of the new drugs is to induce selective transrepression. The development of selectivity in a molecular level may bear less on efficacy.

Tachyphylaxis is the rapidly decreasing response to topical corticosteroids. After corrected and sustained use of topical steroids, the capillaries in the dermis do not constrict as well as before, requiring higher doses or more frequent applications of steroids to achieve the former results. The ability of the blood vessels to constrict as before eventually returns to normal after stopping therapy. The common and known clinical perception of tachyphylaxis may also be significantly related to issues of compliance outside the study group, or to vessels flare unrelated to therapy. Du Vivier and Stoughton, in 1975, were the first describing the persistence and recurrence of psoriasis in patients who were previously treated with topical corticosteroids with a successful result [121]. The question remains if this is a truly clinical entity or if it is just due to a nonadherence to the topical regimen.

Rebound caused by abruptly withdrawal, or ending of steroid therapy by the individual him/herself, can result in sudden worsening of psoriasis. Furthermore, the psoriasis may return more aggressively. A localized or a mild form of psoriasis may become generalized, or a generalized form can be precipitated as pustular or erythrodermic form, when patients do not wean gradually off of corticosteroids. 

The treatment should be tailored in an individual manner, prescribing to each patient the most suitable vehicle. Despite ointments being clinically more effective in psoriasis symptoms, what really matters is the desire of the patient, and the way he/she adheres to the topical treatment.

Each patient will adhere “better” to a different vehicle, some will prefer ointments, others gel or spray, and others will prefer occlusion therapy.

Tight supervision during the treatment with topical corticosteroids by giving support and answers to patient concerns must be provided, and this can make the difference between a successful treatment and a worsening of the disease.

## Figures and Tables

**Table 1:** Corticosteroid classification system, adapted from [12].

Class	Name	Vehicle		
		Oinmtent	Cream	Lotion
Superpotent Class I USA; class I UK; class IV Germany	Betamethasone dipropionate glycol 0.05%			
	Clobetasol 17-propionate 0.05%			
	Halobetasol propionate 0.05%			

High potency Class II/III USA; class II UK; class III Germany	Amcinonide 0.1%			
	Betamethasone dipropionate 0.05%			
	Desoximetasone 0.25%			
	Diflucortolone valerate 0.1%			
	Fluocinonide 0.05%			
	Halcinonide 0.1%			
	Mometasone furoate 0.1%			
	Triamcinolone acetonide 0.5%			

Moderate potency Class IV/V USA; class III UK; class II Germany	Betamethasone dipropionate 0.05%			
	Betamethasone valerate 0.1%			
	Clobetasone 17-butyrate 0.05%			
	Desonide 0.05%			
	Desoximetasone 0.05%			
	Fluocinonide 0.025%			
	Hydrocortisone 17-valerate 0.2%			
	Prednicarbate 0.1%			
	Triamcinolone acetonide 0.1%			

Low potency Class VI/VII USA; class IV UK; class I Germany	Betamethasone valerate 0.05%			
	Desonide 0.05%			
	Fluocinonide 0.01%			
	Hydrocortisone 1.0%, 2.5%			
	Hydrocortisone acetate 0.5%, 1.0%			
	Prednicarbate 0.05%			
	Triamcinolone acetonide 0.025%			
